# Systemic Manifestations of COPD and the Impact of Dual Bronchodilation with Tiotropium/Olodaterol on Cardiac Function and Autonomic Integrity

**DOI:** 10.3390/jcm13102937

**Published:** 2024-05-16

**Authors:** Ieva Dimiene, Deimante Hoppenot, Donatas Vajauskas, Lina Padervinskiene, Airidas Rimkunas, Marius Zemaitis, Diana Barkauskiene, Tomas Lapinskas, Egle Ereminiene, Skaidrius Miliauskas

**Affiliations:** 1 Department of Pulmonology, Medical Academy, Lithuanian University of Health Sciences, 44307 Kaunas, Lithuania; deimante.hoppenot@lsmu.lt (D.H.); marius.zemaitis@lsmu.lt (M.Z.); diana.barkauskiene@lsmu.lt (D.B.); skaidrius.miliauskas@lsmu.lt (S.M.); 2 Department of Radiology, Medical Academy, Lithuanian University of Health Sciences, 44307 Kaunas, Lithuania; donatas.vajauskas@lsmu.lt (D.V.); lina.padervinskiene@lsmu.lt (L.P.); 3 Laboratory of Pulmonology, Department of Pulmonology, Medical Academy, Lithuanian University of Health Sciences, 44307 Kaunas, Lithuania; airidas.rimkunas@lsmu.lt; 4 Department of Cardiology, Medical Academy, Lithuanian University of Health Sciences, 44307 Kaunas, Lithuania; tomas.lapinskas@lsmu.lt (T.L.); egle.ereminiene@lsmu.lt (E.E.)

**Keywords:** chronic obstructive pulmonary disease, systemic inflammation, cardiac dysfunction

## Abstract

**Background**: Chronic obstructive pulmonary disease (COPD) has significant systemic manifestations, including cardiovascular morbidity. The main aim of our study was to evaluate the effect of short-term COPD treatment with tiotropium/olodaterol (TIO/OLO) 5/5 μg on cardiac function and autonomic integrity. **Methods**: Twenty-nine patients with newly diagnosed moderate-to-severe COPD were enrolled. We performed pulmonary function tests, cardiac magnetic resonance, cardiac 123I-metaiodobenzylguanidine (123I-MIBG) imaging and analysis of blood biomarkers on our study subjects. The correlations between the tests’ results were evaluated at baseline. The changes in pulmonary and cardiac parameters from baseline through 12 weeks were assessed. **Results**: Significant associations between pulmonary function tests’ results and high-sensitivity C-reactive protein (hs-CRP), as well as interleukin-22 (IL-22), were observed at baseline. Treatment with TIO/OLO significantly improved lung function as measured by spirometry and body plethysmography. Moreover, we found that the cardiac index increased from 2.89 (interquartile range (IQR) 1.09) to 3.21 L/min/m^2^ (IQR 0.78) (*p* = 0.013; N = 18) and the late heart-to-mediastinum ratio improved from 1.88 (IQR 0.37) to 2 (IQR 0.41) (*p* = 0.026; N = 16) after 12 weeks of treatment. **Conclusions**: Treatment with TIO/OLO improves lung function and positively impacts cardiac function and autonomic integrity, suggesting that dual bronchodilation might have a potential in decreasing the risk for cardiac events in COPD. Hs-CRP and IL-22 might be beneficial in determining the intensity of systemic inflammation in COPD. Further research with a larger cohort is needed to enhance the initial results of this study.

## 1. Introduction

Chronic obstructive pulmonary disease (COPD) is one of the major causes of death globally, with a prediction of becoming the fourth leading cause by 2040 [1]. Even though COPD is a chronic inflammatory respiratory disease, causing airflow limitation and alterations in intrathoracic pressure [2], it also has various systemic impacts, including metabolic, musculoskeletal, hematologic and cardiovascular manifestations [3].

Systemic inflammation, as an important pathophysiological mechanism of COPD, has been thoroughly studied over the past two decades. The impact of particular blood biomarkers (e.g., C-reactive protein (CRP), high-sensitivity (hs)-CRP, interleukin (IL)-6, IL-8, tumor necrosis factor-α, lactate dehydrogenase (LDH), ferritin, D-dimer, neutrophil to lymphocyte ratio (NLR)) on the severity and prognosis of COPD has been widely investigated and described in studies and literature reviews [4,5,6,7,8,9,10]. Certain blood biomarkers, such as natriuretic peptides and troponin, have recently been of great interest in predicting cardiovascular events and mortality in patients with COPD [11,12,13]. Furthermore, new blood biomarkers are being researched to comprehend the role of systemic inflammation in the pathophysiology of COPD [14,15,16].

As COPD shares risk factors with cardiovascular diseases (CVDs) and tends to impair cardiac function through different mechanisms, such as increased intrathoracic pressure, hypoxia, oxidative stress and systemic inflammation [2,17], cardiovascular morbidity is more frequent in people with COPD than in the general population [18]. Moreover, it has been observed that among patients with COPD, cardiovascular morbidities lead to more hospitalizations and deaths than COPD itself [19].

Dual bronchodilation with long-acting β_2_-agonists (LABAs) and long-acting muscarinic antagonists (LAMAs) is still considered the first choice for initial pharmacological therapy for COPD patients with high risk for exacerbations or the ones not responding efficiently to a single bronchodilator [20]. While LABAs and LAMAs are associated with adrenergic and anticholinergic side effects, respectively, the concern that the synergistic effect of LAMA/LABAs combinations might increase the risk for cardiovascular events has been raised [21,22]. However, studies of different models showed that the treatment with LAMA/LABAs combinations did not increase the risk of cardiovascular events comparing to therapies with monocomponents in patients with COPD [23,24,25,26]. Nevertheless, the data comparing COPD treatment with LAMA/LABAs and other inhaled combinations are controversial. For example, a large meta-analysis of clinical trials found that treatment regimens with LAMA/LABAs (including triple therapy) significantly increased the risk for major cardiovascular events, in comparison with combinations of inhaled corticosteroids (ICS) and LABAs [27], while a recent population-based cohort study showed that LAMA/LABAs did not cause a higher risk for cardiovascular events comparing to ICS/LABAs [28].

Since it has been proven that lung hyperinflation is associated with cardiac structural changes and dysfunction [29,30,31,32], it is important to understand whether lung deflation using bronchodilators in patients with COPD might help reducing cardiac impairment. However, only a few studies on the effect of treatment with LAMA/LABAs on cardiac function in COPD patients have been published. The CLAIM study showed that COPD treatment with indacaterol/glycopyrronium (IND/GLY) significantly reduced lung hyperinflation, thus increasing left-ventricular (LV) end-diastolic volume (EDV) by approximately 10% from baseline to 14-day follow-up. The treatment difference was significant in comparison with placebo. These findings were accompanied by significant improvement in other cardiac magnetic resonance imaging (MRI) measurements [33]. The study comparing the effectiveness of tiotropium/olodaterol (TIO/OLO) versus fluticasone/salmeterol (FLU/SAL) on cardiac function in patients with COPD (N = 76) showed that even though TIO/OLO have a higher reduced residual volume (ResV) than that of FLU/SAL, both treatments improved the LV-EDV after six weeks without significant treatment difference [34]. The observational COSYCONET study (N = 846) evaluated the impact of different COPD inhaler therapies over a 1.5-year period on cardiac functional parameters in echocardiography. It was found that treatment with ICS, ICS/LABAs and LAMA/LABAs significantly increased the size of the left atrium by 0.5–0.8 mm. LAMA/LABAs showed the most significant change comparing to other groups. However, no significant improvements were observed in LV end-diastolic diameter, LV end-systolic diameter and LV ejection fraction (EF) during the course of the treatment with different inhalers [35]. According to https://clinicaltrials.gov/, there is one active study evaluating the effect of umeclidinium/vilanterol on cardiac function in patients with COPD; however, no results have been published yet [36].

It has been recognized that COPD has a negative impact on cardiac autonomic regulation [37,38,39]. According to the literature, there are various mechanisms that might lead to impaired autonomic integrity, including increased intrathoracic pressure, recurrent hypoxemia, hypercapnia, and systemic inflammation, as well as the use of beta-sympathomimetics [40]. It has been found that patients with COPD have a lower heart rate recovery (HRR) after exercise, which is a marker of impaired cardiac autonomic integrity and poor prognosis among various populations [41,42,43]. Therefore, it is important that the impairment of cardiac autonomic integrity would be taken into consideration when dealing with COPD patients. As evaluating the changes in HRR during the course of the treatment in these patients might be challenging due to exercise intolerance, comorbidities or aging, other methods requiring fewer physical efforts should be considered. Cardiac 123I-metaiodobenzylguanidine (MIBG) imaging is a non-invasive tool to stratify the risk of sudden arrhythmic death in patients with cardiac diseases [44,45,46,47].

However, little is known about the profile of cardiac adrenergic innervation assessed with cardiac 123I-MIBG imaging in patients with COPD. In the study by Sakamaki et al. it was found that COPD patients had a lower late heart-to-mediastinum ratio (HMR), which is the main predictor of cardiac mortality, than healthy controls (*p* < 0.05). The late HMR did not differ significantly between COPD patients that inhaled β-stimulators or anticholinergic drugs and those that did not [48]. The changes in cardiac adrenergic innervation assessed with cardiac 123I-MIBG imaging during the treatment of COPD with bronchodilators have not been investigated yet.

The main aim of our study was to investigate the effect of short-term treatment with a TIO/OLO fixed-dose combination (FDC) 5/5 µg on pulmonary and cardiac function, as well as cardiac adrenergic innervation. We also sought to evaluate the relationship between blood biomarkers, lung function tests, cardiac functional parameters and cardiac adrenergic innervation in patients with newly diagnosed moderate-to-severe COPD.

## 2. Materials and Methods

### 2.1. Study Subjects

We performed a prospective cohort study that assessed pulmonary function tests (spirometry, body plethysmography and gas diffusion), blood biomarkers (BNP, D-dimer, LDH, NLR, ferritin, hs-CRP and IL-22) and parameters of cardiac MRI as well as cardiac 123I-MIBG imaging in newly diagnosed treatment-naïve moderate-to-severe COPD patients. After 12 weeks of dual bronchodilation with TIO/OLO FDC 5/5 μg, we evaluated the changes in the aforementioned pulmonary function tests and cardiac parameters.

A total of 29 participants were enrolled in this study from September 2021 to December 2023 at the Department of Pulmonology at the Hospital of Lithuanian University of Health Sciences Kauno klinikos. Twenty-two individuals underwent the follow-up investigation after 12 weeks of treatment with TIO/OLO FDC 5/5 μg. Seven were lost to follow-up or could not repeat the investigation due to personal reasons.

Due to technical issues, not all study subjects underwent every investigation. Cardiac 123I-MIBG imaging was conducted on 26 patients at baseline and on 16 of these patients at the 12-week follow-up. Cardiac MRI was performed for 28 patients initially, with 18 undergoing a repeat MRI at 12 weeks. The analysis of IL-22 was carried out for 19 participants, limited by the availability of the test.

The inclusion criteria for this study were as follows: individuals aged 40 years and older, with a newly diagnosed case of moderate-to-severe COPD (defined by forced expiratory volume in 1 s (FEV_1_) and forced vital capacity (FVC) ratio (FEV_1_/FVC) < 70% and FEV1 between 30 to 79% of the predicted value), a smoking history of at least 10 pack years, and being able to sign informed consent. We excluded patients with any of the following conditions: uncontrolled arterial hypertension, current arrhythmias, interstitial lung diseases, lung cancer, documented hypercapnia, dementia, pregnancy, a history of asthma, or confirmed alpha-1 antitrypsin deficiency. Having a stable cardiovascular morbidity controlled with medications (e.g., statins, antihypertensive drugs, diuretics, antiplatelet drugs) was not considered an exclusion criterion. However, in order to reduce the impact of other medications on the study results, patients who had adjustments to the treatment of their concomitant diseases within one month prior to or during the study period were excluded.

This study was authorized by the Kaunas Regional Biomedical Research Ethics Committee (no. BE-2-46, 14 April 2021, Kaunas, Lithuania). This study was registered in the National Institutes of Health trial registry of United States https://clinicaltrials.gov/ with the identification number NCT06072690.

### 2.2. Blood Biomarkers’ Testing

Seven blood biomarkers were tested in this study: BNP, D-dimer, LDH, NLR, ferritin, hs-CRP, and IL-22. The IL-22 levels were quantified using an enzyme-linked immunosorbent assay (ELISA) with the IL-22 Human ELISA Kit (Invitrogen™, Waltham, MA, USA), following the manufacturer’s instructions. This assay was conducted in the Laboratory of Pulmonology at the Department of Pulmonology, Lithuanian University of Health Sciences. The other blood biomarkers were analyzed in the laboratory of the Hospital of Lithuanian University of Health Sciences Kauno Klinikos, adhering to the established local laboratory protocols.

### 2.3. Pulmonary Function Tests

FEV_1_, FVC and FEV_1_/FVC were measured with a Ganshorn spirometry device (Ganshorn Medizin Electronic, Niederlauer, Germany). Body plethysmography was performed in a Ganshorn Power Cube Body+ plethysmography chamber (Ganshorn Medizin Electronic, Niederlauer, Germany). The following parameters were assessed (individual values in L or % (depending on parameter) and % of predicted values (% pred.)): functional residual capacity (FRC), ResV, total lung capacity (TLC), FRC-to-TLC ratio (FRC/TLC) and ResV-to-TLC ratio (ResV/TLC). Gas diffusion was performed in Ganshorn PowerCube Diffusion+ system (Ganshorn Medizin Electronic, Niederlauer, Germany). We assessed the diffusing capacity of the lungs adjusted for hemoglobin level (DLCOc). The values are described as mmol/min/kPa or % pred.

Pulmonary function tests were performed and interpreted according to recent European Respiratory Society (ERS) and American Thoracic Society (ATS) guidelines [49].

### 2.4. Cardiac Magnetic Resonance Imaging

Cardiac MRI scans were performed using a 3.0 T whole-body system (Siemens Skyra, Siemens Medical Solutions; Erlangen, Germany), according to local protocol approved by the Hospital of Lithuanian University of Health Sciences Kauno klinikos. The analysis of imaging was performed using commercial software Medis (QMass 8.1, version 4.0.62.4, Medical Imaging System, Leiden, The Netherlands). Biventricular evaluations of EDV and end-systolic volume (ESV) were performed. The stroke volume (SV) and EF of both ventricles were calculated from the EDV and ESV. We calculated LV cardiac mass (LV-CM) by summing the slices, manually tracing the epicardial and endocardial borders in each end-diastolic slice, and further multiplying by the thickness of the slice in order to compute the myocardial volume. All the cardiac volumes and LV-CM were indexed for body surface area (i). The cardiac index (CI) was obtained in our analysis, too. It was described as the cardiac output (SV multiplied by the heart rate) divided by body surface area.

### 2.5. Cardiac 123I-Metaiodobenzylguanidine Imaging

During this investigation, the patients had a two-dimensional anterior chest scan and cardiac single-photon emission computed tomography (SPECT) scan performed 15 min (early scan) and 4 h (late scan) after an intravenous injection of 123I-MIBG solution (Iobeguane I123 GE healthcare) using a dual-detector Mediso AnyScan SC gamma camera (Mediso, Budapest, Hungary) for planar imaging acquisition and a GE Discovery NM 530c dedicated cardiac gamma camera (GE Health Care, Boston, MA, USA) for SPECT imaging acquisition. Early and late planar images were acquired 15 min and 4 h after the 123I-MIBG injection using low-energy general purpose collimators and 128 × 128 matrix—5 min acquisition. Early and late cardiac SPECT images were acquired straight after planar images using a dedicated cardiac system—7 min acquisition. HMRs were obtained by manually drawing the regions of interest of the heart (H) and mediastinum (M) in early and propagated on late two-dimensional anterior chest scans and setting the ratio. The washout rate (WR) was calculated using the formula in Equation (1).

(1) $$\mathrm{WR}=\frac{\left(\mathrm{Early}\;H-\mathrm{Early}\;M\right)-\left(\mathrm{Late}\;H-\mathrm{Late}\;M\right)\times \mathrm{DCF}}{\mathrm{Early}\;H\;-\mathrm{Early}\;M}\times 100\;\left(\%\right)$$

Calculation of washout rate (WR) with decay correction factor (DCF) 1.21. Abbreviations: H—heart count, M—mediastinum count.

Cardiac SPECT scans were reconstructed and early as well as late LV total defect scores (TDS) were evaluated. The patients’ preparation for cardiac 123I-MIBG imaging, and the investigation and the evaluation of results were performed according to European Association of Nuclear Medicine (EANM) guidelines [50].

### 2.6. Interventions and Follow-Up

After undergoing baseline examinations, including sampling of blood biomarkers, spirometry, body plethysmography, gas diffusion tests, cardiac MRI and cardiac 123I-MIBG imaging, the subjects were instructed to return for a follow-up visit after 12 weeks of treatment with TIO/OLO FDC at a dose of 5/5 μg to repeat pulmonary and cardiac investigations. Treatment compliance was verified by reviewing electronic health records, ensuring adherence to the prescribed regimen based on prescription fill records and patient confirmations.

### 2.7. Statistical Analysis

Statistical analyses were conducted using IBM SPSS Statistics for Windows, version 21.0 (IBM Corp., Armonk, NY, USA). We analyzed the data from patients newly diagnosed with moderate-to-severe COPD, both before and after 12 weeks of treatment with TIO/OLO FDC 5/5 μg. Due to the small size of our study cohort, we did not categorize patients into different COPD GOLD stages for our analyses. To assess the strength and direction of associations between pulmonary function tests, blood biomarkers, and results from cardiac MRI and cardiac 123I-MIBG imaging before the treatment, we employed Spearman’s correlation analysis. The Wilcoxon signed-rank test, a non-parametric method, was used to evaluate the significance of changes in the measured parameters from baseline to the 12-week follow-up. The results were deemed statistically significant at *p* ≤ 0.05.

## 3. Results

### 3.1. Patients’ Characteristics

In this study, we analyzed a cohort of 29 newly diagnosed patients with moderate-to-severe COPD (GOLD stages 2 and 3), aged between 40 and 76 years. In our study cohort, moderate COPD (GOLD 2) was dominant over severe (GOLD 3) (N = 21 vs. N = 8, respectively). The majority of patients had characteristics of air trapping (N = 6) or lung hyperinflation (N = 16) in body plethysmography. Thirteen patients had impaired gas diffusion, and three were borderline. Twenty-three subjects had features of lung emphysema in the chest computed tomography scan. All studied patients had normal or clinically insignificantly reduced LV-EF (median 59.5 (%); IQR 11.5) as measured by cardiac MRI but no history of congestive heart failure. The most common concomitant diseases were arterial hypertension and dyslipidemia. The main demographic and clinical characteristics of the study cohort are detailed in Table 1, while the median values of the baseline pulmonary function tests are presented in Table 2.

### 3.2. Correlations between Blood Biomarkers and Pulmonary Function Tests at Baseline

The median values of blood biomarkers at baseline are presented in Table 3.

**Figure 1:**
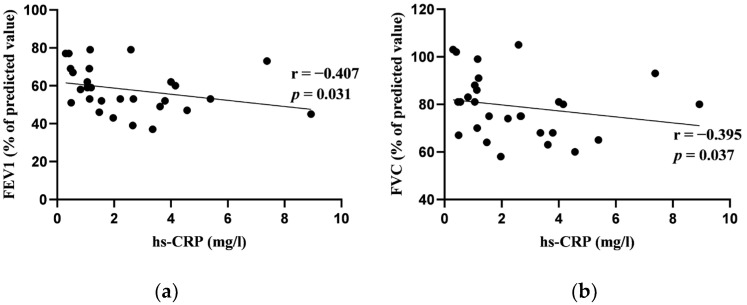
(**a**) Correlation between high-sensitivity C-reactive protein (hs-CRP) and forced expiratory volume in 1 s (FEV_1_); (**b**) correlation between hs-CRP and forced vital capacity (FVC) at baseline.

**Figure 2:**
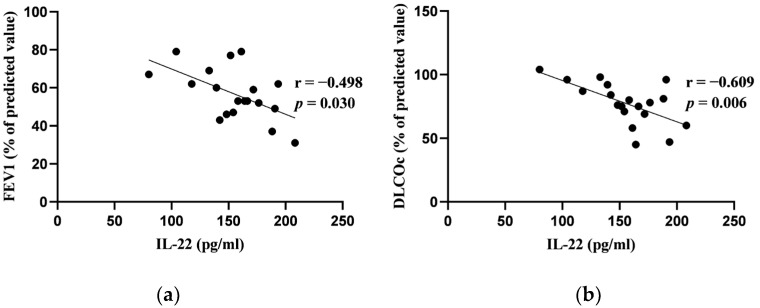
(**a**) Correlation between interleukin-22 (IL-22) and forced expiratory volume in 1 s (FEV_1_); (**b**) correlation between IL-22 and diffusing capacity of the lungs adjusted for hemoglobin level (DLCOc) at baseline.

In our study, among the blood biomarkers analyzed, only hs-CRP and IL-22 showed significant correlations with the results of the pulmonary function tests at baseline. Specifically, hs-CRP correlated negatively with both FEV_1_ (% pred.) and FVC (% pred.) at baseline. Similarly, IL-22 exhibited a negative correlation with FEV_1_ (% pred.) and DLCOc (%). Other blood biomarkers (D-dimer, ferritin, LDH, NLR, BNP) had no significant correlations with pulmonary function tests. Figure 1 and Figure 2 depict these significant correlations.

### 3.3. Correlations between Blood Biomarkers and Cardiac Functional Parameters

We observed that BNP demonstrated significant negative correlations with both LV-ESVi (*p* = 0.047; r = −0.379) and right ventricular (RV) ESVi (*p* = 0.037; r = −0.396) as measured by cardiac MRI at baseline. There was also a trend towards a negative correlation between BNP and HMRs (early and late) (*p* = 0.06; r = −0.373 and *p* = 0.067; r = −0.365, respectively). These trends did not reach statistical significance, though. No significant correlations were detected between other blood biomarkers and cardiac functional parameters.

### 3.4. Correlations between Cardiac Magnetic Resonance Imaging Parameters and Pulmonary Function Tests at Baseline

The median values of cardiac MRI parameters at baseline are presented in Table 4.

We discovered significant correlations between pulmonary function tests’ results and several left-sided heart parameters at baseline, including LV-EF, LV-EDVi, LV-ESVi, LV-SVi, and CI. Specifically, LV-EF negatively correlated with both FRC (% pred.) and FRC (L), and showed a tendency towards a negative correlation with ResV (% pred.) LV-EDVi demonstrated a negative correlation with ResV/TLC (%) and a tendency towards a positive correlation with FEV_1_ (L). A positive correlation was observed between LV-ESVi and FVC (L). LV-SVi showed a negative correlation with ResV/TLC (%). CI correlated negatively with ResV/TLC (%) and positively with FVC (L), with a tendency towards a positive correlation with FEV_1_ (L). These findings are detailed in Table 5.

We found significant correlations between right-sided heart parameters and pulmonary function tests. RV-EDVi correlated negatively with ResV/TLC (%) and positively with FEV_1_ (L), as well as FVC (L). RV-SVi had a positive correlation with FEV_1_ (L). RV-EF showed a tendency of positive correlation with FEV_1_ (%). The correlations are presented in Table 6. No other significant correlations were found between measured cardiac MRI parameters and the results of pulmonary function tests.

### 3.5. Correlations between Cardiac 123I-Metaiodobenzylguanidine Imaging Adrenergic Innervation Parameters and Pulmonary Function Tests at Baseline

The median values of cardiac 123I-MIBG adrenergic innervation parameters at baseline are presented in Table 7.

**Figure 3:**
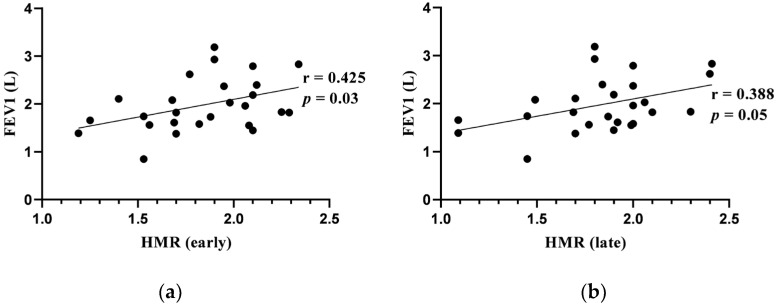
(**a**) Correlation between early heart-to-mediastinum ratio (HMR) and forced expiratory volume in 1 s (FEV1) at baseline; (**b**) correlation between late HMR and forced expiratory volume in 1 s (FEV1) at baseline.

**Figure 4:**
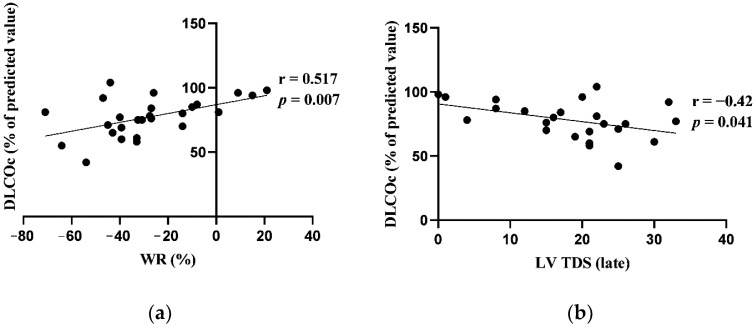
(**a**) Correlation between washout rate (WR) and diffusing capacity of the lungs adjusted for hemoglobin level (DLCOc) at baseline; (**b**) correlation between late left ventricular (LV) total defect score (TDS) and DLCOc at baseline.

Early and late HMRs correlated positively with FEV_1_ (L). There was a significant positive correlation between WR and DLCOc (% pred.). Late LV TDS correlated negatively with DLCOc (% pred.) No other significant correlations were found between evaluated cardiac 123I-MIBG imaging adrenergic innervation parameters and the results of pulmonary function tests. Significant correlations are presented in Figure 3 and Figure 4.

### 3.6. Changes in Pulmonary Function Tests from Baseline to 12-Week Follow-Up

After 12 weeks of treatment with TIO/OLO FDC 5/5 μg, a significant improvement in FEV_1_ was observed. Significant reductions in ResV/TLC and FRC/TLC (% pred.) were also noted in the body plethysmography results. However, the gas diffusion measurements did not show significant changes when compared to the baseline values (Table 8).

### 3.7. Changes in Cardiac Magnetic Resonance Imaging Parameters from Baseline to 12-Week Follow-Up

The analysis of cardiac MRI data from 18 patients, conducted at baseline and following 12 weeks of treatment with TIO/OLO FDC 5/5 μg, revealed an improvement in the CI from 2.89 (IQR 1.09) to 3.21 (IQR 0.78) (*p* = 0.013). Other cardiac MRI parameters did not show significant changes during the study period.

### 3.8. Changes in Cardiac 123I-Metaiodobenzylguanidine Imaging Adrenergic Innervation Parameters from Baseline to 12-Week Follow-Up

The cardiac 123I-MIBG imaging results for 16 patients, assessed at baseline and again at the 12-week follow-up, indicated a statistically significant improvement in the late HMR. No significant changes were observed in other cardiac 123I-MIBG imaging parameters, as detailed in Table 9.

## 4. Discussion

### 4.1. Blood Biomarkers

We found that only two out of seven studied blood biomarkers (hs-CRP and IL-22) were significantly correlated with the results of pulmonary function tests at baseline. Moderate inverse correlations between hs-CRP and FEV_1_ as well as FVC were observed in our subjects. Greater serum IL-22 concentrations were associated with a reduction in FEV_1_ and DLCOc. Previous research showed that the levels of hs-CRP were significantly higher in patients with COPD than healthy controls. The mean values of hs-CRP varied from 0.88 to 3.14 mg/l in healthy subjects and from 4.82 to 7.22 mg/L in COPD patients [51,52,53]. However, the data regarding the relation between CRP or hs-CRP and pulmonary function tests in COPD are controversial. Some studies did not find significant correlations between hs-CRP and spirometry results in stable COPD patients [10,51]. Nevertheless, there are publications showing that hs-CRP plays an important role in airflow limitation in COPD. Aksu et al. found significant negative correlations between hs-CRP and FEV_1_ % pred., as well as FVC % pred. [52]. Agarwal et al. found that hs-CRP had a strong negative correlation between hs-CRP and FEV_1_ [53]. Both studies included patients of GOLD 1 to 4 COPD stages. It has been suggested that one of the main mechanisms through which CRP is related to COPD is a persisting lung inflammation that activates systemic response, thus promoting the respiratory inflammation [54]. Our data support this hypothesis showing that even mild elevation in hs-CRP concentration is associated with greater airflow limitation. As increased CRP levels in patients with stable COPD are associated with poor outcomes, including the occurrence of cardiovascular and thrombotic events, CRP assessment should be taken into consideration when COPD is newly diagnosed [55,56].

Recently, increasing attention has been paid to the role of various cytokines, including IL-22, in systemic inflammation in COPD. IL-22 is produced by various lymphoid cells, including Th1, Th17 and Th22, innate lymphoid cells, and natural killer T cells, as well as non-lymphoid cells (fibroblasts, macrophages, mast cells, neutrophils), depending on the disease [57]. However, the data regarding the role of blood IL-22 in COPD are scarce. Xiong et al. found that plasma IL-22 levels did not differ significantly between healthy non-smoking controls (HC), healthy smokers (HS), stable and exacerbated COPD [58]. Another study revealed that patients with stable COPD and HS had higher serum IL-22 levels than HC (*p* < 0.01) with the mean serum IL-22 in HC group below 100 pg/mL. However, the concentration of serum IL-22 did not correlate with pulmonary function tests in COPD patients [59]. In our study, higher serum IL-22 concentration was significantly associated with a reduction in FEV_1_. This could indicate that there might be a relation between serum IL-22 and the severity of COPD; however, a study with a larger cohort should be performed. We also observed that higher serum IL-22 levels were inversely related to lower DLCOc. In accessible databases, no studies evaluating the relationship between IL-22 and gas diffusion in patients with COPD were found. As decreased DLCOc is associated with alveolar destruction and emphysema due to airway inflammation in COPD, our study findings might be explained by the trigger of a systemic response due to an inflammatory process in the alveoli [60,61,62].

Regardless of the data from previous studies revealing that the rest of the investigated blood biomarkers (e.g., ferritin, D-dimer, BNP, NLR, LDH) were associated with lung function impairment, we did not find any significant relationship between the aforementioned assays and pulmonary function measurements [9,63,64,65,66]. However, that might be related to the small size of our study group.

In our research, we found significant correlations between BNP and cardiac function measured by MRI. Moderately negative correlations between RV-ESVi, LV-ESVi and BNP were observed. However, these results might appear inconsistent with the previously published data. BNP is predominantly produced by cardiomyocytes due to myocardial wall stress and its increase is related to the overload of cardiac volumes in various cardiac pathologies [67,68,69]. We assume that the findings in our subjects might be associated with repeated intermittent mild hypoxia, which stimulates the production of natriuretic peptides and plays an important role in the development of cardiac structural changes [70,71]. A significant inverse moderate correlation between BNP and HMR in cardiac 123I-MIBG imaging was observed in patients with congestive heart failure in previous studies [72,73]. Despite the absence of studies exploring the relationship between BNP levels and cardiac 123I-MIBG imaging parameters in COPD patients without a known history of congestive heart failure, our findings suggest a trend towards a negative correlation between BNP levels and HMR, both early and late, although these did not achieve statistical significance. This highlights the potential value of investigating this relationship further in a larger cohort, which might yield more definitive results.

### 4.2. Pulmonary Function Tests

In our study, a significant improvement in lung function was observed after 12 weeks of COPD treatment with TIO/OLO FDC 5/5 μg. The treatment significantly improved FEV_1_ and lowered the values describing air-trapping and lung hyperinflation (ResV/TLC and FRC/TLC). A large meta-analysis of 22 studies (N = 16 486) showed that COPD treatment with various LAMA/LABAs increased FEV_1_ from 119 to 185 mL after 2 days of treatment compared to baseline values. Particularly, the treatment with TIO/OLO increased FEV_1_ by 0.170 (95% CI: 0.121–0.220 L). However, the majority of LAMA/LABAs, including TIO/OLO, showed a reduction in efficacy at 24 weeks. The increase in FEV_1_ from baseline at 24 weeks for TIO/OLO was of 0.129 (95% CI: 0.087–0.172) [74]. OTEMTO 1 and 2 trials were not included in this meta-analysis, though. In these studies (N = 406), it was found that after 12 weeks of COPD treatment with TIO/OLO FDC 5/5 μg, the FEV_1_ area under the curve from 0 to 3 h response improved in 0.331 L and 0.299 L in OTEMTO 1 and 2, respectively. The change was significant in comparison with placebo [75]. In our small study cohort, the median change in FEV_1_ after 12 weeks of treatment with TIO/OLO FDC 5/5 μg was similar—310 mL (*p* < 0.0002) (the median change in FEV_1_ % pred. was 6.5%). It is important to mention that our patients had their TIO/OLO FDC 5/5 μg inhalations 2 h before the pulmonary function tests during the 12-week follow-up visit.

According to previous research, short-term COPD treatment with different LAMA/LABAs significantly improved body plethysmography results, including ResV, FRC, inspiratory capacity (IC), airway resistance (Raw) [33,76,77,78]. COPD treatment with TIO/OLO FDC 5/5 μg for six weeks significantly reduced FRC and ResV in VIVACITO study. FRC reduced by 547 mL and ResV by approximately 700 mL from baseline to six-week follow-up at 2 h 30 min post-dose in patients treated with TIO/OLO. These results differed significantly from those of the placebo group [79]. In our study, we found that TIO/OLO FDC 5/5 μg significantly improved ResV/TLC (reduction in % pred. was 11%; reduction in individual value was 4.33%) and FRC/TLC (reduction in % pred. was 3.5%).

The improvement in FEV_1_ caused by TIO/OLO FDC 5/5 μg in our study supports the conclusion that this combination is an effective treatment option for moderate-to-severe stable COPD patients. However, we consider that changes in body plethysmography results might have been more significant in our study if all patients had increased ResV/TLC and (or) FRC/TLC at baseline.

### 4.3. Cardiac Magnetic Resonance Imaging

The results of our study show that LV and RV function is significantly affected by airflow limitation and the changes in intrathoracic pressures in patients with COPD. Earlier research revealed that increased intrathoracic pressure plays a crucial role in the development of cardiac structural changes and dysfunction in COPD [29,30,80]. It has been estimated that COPD patients (even those without cardiovascular diseases) have significantly lower heart volumes than healthy controls. The main reason for that is the reduction in the preload caused by intrathoracic hypovolemia due to lung hyperinflation [81,82]. Moreover, hypoxemia, oxidative stress and systemic inflammatory response aggravate the damage of cardiovascular system in COPD patients [83]. Another mechanism for cardiac deterioration is called interdependence and it is present in advanced COPD when the RV dysfunction leads to the impairment of LV function [84]. Nevertheless, we assume that this was not a case in our patient cohort, where subjects of GOLD 2–3 stages were selected.

The cardiovascular safety of LAMA/LABAs use in patients with COPD has been confirmed by various trials and large meta-analyses [28,85,86,87,88,89,90]. However, there are little data regarding their positive effects on cardiac function. Two studies that evaluated the effect of LAMA/LABAs on cardiac functional changes measured by MRI have been published to date. In our study, a significant increase in CI from median 2.89 L/min/m^2^ (IQR 1.09) at baseline to 3.21 L/min/m^2^ (IQR 0.78) at 12-week follow-up was observed. No other significant changes from baseline to 12 weeks follow-up in cardiac parameters as measured by MRI were found. A significant improvement in CI during the treatment with other LAMA/LABA (IND/GLY) was observed in the CLAIM study (N = 30 ING/GLY vs. N = 32 placebo) as well. CI changed from mean 2.32 L/min/m^2^ (SD 0.58) at baseline to the least-squares mean 2.59 L/min/m^2^ (95% CI 2.43–2.75) at 14 days follow-up; the results were significantly better compared to those of the placebo. Furthermore, significant changes in biventricular volumes from baseline to 14 days follow-up were observed in IND/GLY group [33]. The prospective study by Herth et al. (N = 76) revealed that the mean LV-EDVi in cardiac MRI significantly increased by 2.317 mL/m^2^ (95% CI 0.061–4.574) after six weeks of COPD treatment with TIO/OLO 5/5 µg. No other significant changes in cardiac functional parameters were observed [34]. We assume that more significant changes in cardiac parameters could have been obtained in our study if lung hyperinflation was one of the inclusion criteria.

### 4.4. Cardiac 123I-Metaiodobenzylguanidine Imaging

Significant associations between cardiac 123I-MIBG adrenergic innervation activity and lung function tests were found in our study. Early and late HMR had moderately positive correlations with FEV_1_ at baseline. There were moderately positive correlations between DLCOc and WR, as well as LV-TDS. In a study by Sakamaki et al., where the cardiac 123I-MIBG profile in COPD patients (N = 28) and healthy volunteers (N = 7) was evaluated, it was found that WR significantly moderately correlated with FVC, ResV/TLC and maximal voluntary ventilation (MVV). No significant correlations between WR and FEV_1_ or DLCOc were observed. In this study, HMR did not correlate with pulmonary function measurements [48]. The data from both studies show that the cardiac 123I-MIBG activity is affected by airflow limitation and intrathoracic pressure variations in patients with COPD. Decreased cardiac adrenergic innervation parameters assessed with cardiac 123I-MIBG imaging represents increased adrenergic overdrive in patients with COPD.

According to the meta-analysis of seven studies with cardiac diseases, the most powerful predictor for cardiac mortality in 123I-MIBG imaging is reduced late HMR with threshold values between 1.5 and 1.89. Patients with values above the threshold had a better prognosis compared with those with values below the threshold (2-year mortality rate 0.02–0.11 vs. 0.12–0.56, accordingly). The thresholds for WR indicating a greater mortality were between 38 and 53%, with a higher percentage indicating a worse prognosis (2-year mortality rate 0.11–0.64 above vs. 0.01–0.15 below the cutoff value) [91]. Another study found that increased LV TDS with the threshold ranging from 37 to 39 in cardiac 123I-MIBG imaging was significantly associated with the development of cardiac arrhythmias during cardiac electrophysiology testing (the area under the ROC curve was 0.76). This finding shows that late LV TDS might also be used as a predictor of cardiac events [92].

The median late HMR in our subjects at baseline was 1.89 (IQR 0.30; N = 26). In the previously mentioned study by Sakamaki et al., COPD patients had similar mean late HMR—1.98 (SD 0.62; N = 28) [48]. According to previously published data, late HMRs in healthy patients were greater, with values varying from 2.38 (SD 0.29) to 2.49 (SD 0.16) [48,93]. However, we understand that lower late HMR in our subjects might not only be associated with impaired autonomic integrity due to COPD, as we did not exclude patients with stable (or undiagnosed asymptomatic) ischemic heart disease from our study. Other cardiac 123I-MIBG adrenergic innervation parameters (WR and LV TDS) did not exceed the previously suggested thresholds for worse cardiac prognosis in our subjects at baseline.

The key finding in our study was a significant increase in late HMR in cardiac 123I-MIBG imaging from baseline to 12 weeks follow-up (from median 1.88 (IQR 0.37) to 2 (IQR 0.41), *p* = 0.026, N = 16), representing a reduction in cardiac adrenergic overdrive. We did not find any studies that evaluated the changes in cardiac 123I-MIBG activity in COPD patients in the course of the treatment with inhalers. However, Sakamaki et al. found no significant difference in late HMR among patients that inhaled anticholinergic drugs (mean HMR, 2.17 (SD 0.51); N = 16) or β-stimulators (mean HMR, 1.93 (SD 0.48); N = 17) and those that did not (mean HMR, 1.89 (SD 10.3); N = 12) [48]. Regarding our study’s data, we consider that COPD treatment with dual bronchodilation improves cardiac autonomic integrity, probably by reducing airflow limitation and intrathoracic pressure. Moreover, as late HMR is a strong predictor for cardiovascular events, COPD treatment with TIO/OLO FDC 5/5 μg might have potential in reducing the risk for sudden cardiac death; however, a longer study period is required to establish the risk for cardiovascular events in these patients.

## 5. Limitations of this Study

This study has several limitations that warrant mentioning. Firstly, the relatively small sample size, while sufficient for detecting the primary outcomes as significant, may limit the generalizability of our findings and the detection of subtler effects. Future research with larger cohorts is planned to build upon these initial results. Secondly, the absence of a control group precludes direct comparisons between COPD patients and healthy individuals, which could provide additional insights into the baseline characteristics and treatment effects. Although our primary objective was to assess treatment efficacy, the inclusion of a control group in future studies could enrich our understanding. Lastly, the exclusion of air trapping and lung hyperinflation as specific inclusion criteria may have constrained our ability to observe more pronounced improvements in cardiac function. Future studies that specifically include patients with these characteristics might yield more significant findings regarding cardiac function improvements in COPD patients.

## 6. Conclusions

Treatment with TIO/OLO 5/5 µg has been shown to significantly improve pulmonary function, which, in turn, positively impacts cardiac function and autonomic integrity. As impaired cardiac autonomic integrity is associated with an increased risk of arrhythmias and sudden cardiac death, our findings suggest that COPD treatment with LAMA/LABAs might have a potential in decreasing the risk for cardiac events in these patients. Blood biomarkers, such as hs-CRP and IL-22, might be beneficial in determining the intensity of systemic inflammation in COPD. Further research with a larger cohort is needed to enhance the initial results of our study.

## Figures and Tables

**Table 1:** Demographic characteristics of study patients and concomitant diseases.

Characteristic	Value
Age (years), median (IQR)	60 (10)
BMI (kg/m^2^), median (IQR)	26.5 (6.6)
Smoking history (pack years), median (IQR)	40 (30)
Gender, n (%) Male Female	26 (89.66) 3 (10.34)
Severity of COPD (airflow limitation), n (%) Moderate (GOLD 2) Severe (GOLD 3)	21 (72.4) 8 (27.6)
Emphysema, n (%)	23 (79.3)
Concomitant diseases, n (%) Arterial hypertension Dyslipidemia Ischemic heart disease Type 2 diabetes mellitus Arrhythmias in the past Hyperuricemia	19 (65.5) 14 (48.3%) 4 (13.8%) 1 (3.4%) 2 (6.9%) 3 (10.3%)

Abbreviations: IQR—interquartile range, BMI—body-mass index, COPD—chronic obstructive pulmonary disease, GOLD—Global Initiative for Chronic Obstructive Lung Disease.

**Table 2:** The median values of baseline pulmonary function tests.

Pulmonary Function Parameter (N = 29)	Baseline Median (IQR)
FEV_1_, % pred. FEV_1_, L	53 (20) 1.83 (0.79)
FVC, % pred. FVC, L	80 (19.5) 3.4 (0.92)
FRC, % pred. FRC, L	123 (35) 4.2 (1.37)
ResV, % pred. ResV, L	145 (40) 3.6 (1.05)
ResV/TLC, % pred. ResV/TLC, %	130 (17) 51.8 (7.87)
FRC/TLC, % pred. FRC/TLC, %	118 (22) 61.08 (9.58)
TLC, % pred. TLC, L	100 (20) 6.97 (1.69)
DLCOc, % pred. DLCOc, mmol/min/kPa	76 (23) 6.66 (3.14)

Abbreviations: IQR—interquartile range, FEV_1_—forced expiratory volume in 1 s, FVC—forced vital capacity, ResV—residual volume, ResV/TLC—residual-volume-to-total-lung-capacity ratio, FRC/TLC—functional-residual-capacity-to-total-lung-capacity ratio, TLC—total lung capacity, DLCOc—diffusing capacity of the lungs adjusted for hemoglobin level, % pred.—percentage of predicted value.

**Table 3:** The median values of blood biomarkers at baseline.

Blood Biomarker (N = 29)	Baseline Median (IQR)
BNP (ng/L)	14.4 (15.1)
LDH (U/L)	224 (46)
Ferritin (μg/L)	87 (111.8)
D-dimer (mg/L)	0.65 (0.45)
Hs-CRP (mg/L)	1.77 (2.69)
NLR	2.14 (1.01)
IL-22 (N = 19) (pg/mL)	158.24 (37.06)

Abbreviations: IQR—interquartile range, BNP—brain natriuretic peptide, LDH—lactate dehydrogenase, Hs-CRP—high-sensitivity C-reactive protein, NLR—neutrophil-to-lymphocyte ratio, IL-22—interleukin-22.

**Table 4:** The median values of cardiac magnetic resonance imaging parameters at baseline.

Cardiac MRI Parameters (N = 28)	Baseline Median (IQR)
LV-EDVi (mL/m^2^)	72.19 (30.04)
RV-EDVi (mL/m^2^)	71.5 (22.64)
LV-ESVi (mL/m^2^)	30.5 (15.05)
RV-ESVi (mL/m^2^)	29 (21.43)
LV-EF (%)	59.5 (11.5)
RV-EF (%)	59 (10.5)
CI (L/min/m^2^)	2.89 (0.99)
LV-SVi (mL/m^2^)	44.06 (17.75)
RV-SVi (mL/m^2^)	42.92 (15)
LV-CMi (g/m^2^)	70 (19.5)

Abbreviations: IQR—interquartile range, LV—left ventricular, RV—right ventricular, EF—ejection fraction, EDV—end-diastolic volume, ESV—end-systolic volume, SV—stroke volume, CM—cardiac mass, i—indexed to body surface area, CI—cardiac index.

**Table 5:** Correlations between left-sided heart function parameters in cardiac magnetic resonance imaging and pulmonary function tests at baseline.

Cardiac MRI	Pulmonary Function Tests	Correlation Coefficient (r)	*p*-Value *
LV-EF, %	FRC, % pred.	−0.378	0.047
	FRC, L	−0.413	0.029
	ResV, % pred.	−0.36	0.06
LV-EDVi, ml/m^2^	ResV/TLC, %	−0.406	0.032
	FEV_1_, L	0.371	0.052
LV-ESVi, ml/m^2^	FVC, L	0.416	0.028
LV-SVi, ml/m^2^	ResV/TLC, %	−0.442	0.018
CI, L/min/m^2^	ResV/TLC, %	−0.586	0.001
	FVC, L	0.388	0.041
	FEV_1_, L	0.354	0.064

* *p*-value ≤ 0.05 indicates statistically significant results. Abbreviations: MRI—magnetic resonance imaging, LV—left ventricular, EF—ejection fraction, EDV—end-diastolic volume, ESV—end-systolic volume, SV—stroke volume, i—value indexed to body surface area, CI—cardiac index, FRC—functional residual capacity, ResV—residual volume, ResV/TLC—residual-volume-to-total-lung-capacity ratio, FEV_1_—forced expiratory volume in 1 s, FVC—forced vital capacity, % pred.—percentage of predicted value.

**Table 6:** Correlations between right-sided heart function parameters in cardiac magnetic resonance imaging and pulmonary function tests at baseline.

Cardiac MRI	Pulmonary Function Tests	Correlation Coefficient (r)	*p*-Value *
RV-EDVi, mL/m^2^	ResV/TLC, %	−0.394	0.038
	FEV_1_, L	0.416	0.028
	FVC, L	0.388	0.041
RV-SVi, mL/m^2^	FEV_1_, L	0.413	0.029
RV-EF, %	FEV_1_, % pred.	0.370	0.052

* *p*-value ≤ 0.05 indicates statistically significant results. Abbreviations: MRI—magnetic resonance imaging, RV—right ventricular, EDV—end-diastolic volume, SV—stroke volume, i—value indexed to body surface area, EF—ejection fraction, ResV/TLC—residual-volume-to-total-lung-capacity ratio, FEV_1_—forced expiratory volume in 1 s, FVC—forced vital capacity, % pred.—percentage of predicted value.

**Table 7:** The median values of cardiac 123I-metaiodobenzylguanidine parameters at baseline.

Cardiac 123I-MIBG Parameter (N = 26)	Baseline Median (IQR)
HMR (early)	1.89 (0.45)
HMR (late)	1.89 (0.30)
WR (%)	31.65 (30.3)
LV TDS (early)	11 (13)
LV TDS (late)	20.5 (12)

Abbreviations: IQR—interquartile range, HMR—heart-to-mediastinum ratio, WR—washout rate, LV—left ventricular, TDS—total defect score.

**Table 8:** Changes in pulmonary function tests from baseline to 12-week follow-up.

N = 22	Baseline Median (IQR)	12 Weeks Follow-Up Median (IQR)	*p*-Value *
FEV_1_, % pred. FEV_1_, L	56 (20.5) 1.84 (1.01)	62.5 (23) 2.15 (1)	0.000124 0.000139
FVC, % pred. FVC, L	77.5 (21.75) 3.39 (1.14)	77 (24.25) 3.41 (0.91)	0.356 0.179
FRC, %	117.5 (46)	113.5 (40)	0.149
FRC, L	4.14 (1.84)	4.05 (1.08)	0.179
ResV, % pred. ResV, L	152 (55) 3.62 (1.25)	136 (37.25) 3.38 (0.96)	0.093 0.276
ResV/TLC, % pred. ResV/TLC, %	130.5 (28) 50.33 (11.08)	119.5 (18.25) 46 (9.81)	0.012 0.017
FRC/TLC, % pred. FRC/TLC, %	116.5 (23) 60.03 (9.89)	113 (17) 57.79 (10.28)	0.047 0.079
TLC, % pred. TLC, L	100 (29) 6.95 (1.99)	100 (17) 7.02 (1.42)	0.981 0.88
DLCOc, % pred. DLCOc, mmol/min/kPa	77 (21.25) 6.85 (3.17)	80 (24.5) 7.35 (3.79)	0.511 0.452

* *p*-value ≤ 0.05 indicates statistically significant results. Abbreviations: FEV_1_—forced expiratory volume in 1 s, FVC—forced vital capacity, FRC—functional-residual-capacity, ResV—residual volume, ResV/TLC—residual-volume-to-total-lung-capacity ratio, FRC/TLC—functional-residual-capacity-to-total-lung-capacity ratio, TLC—total lung capacity, DLCOc—diffusing capacity of the lungs adjusted for hemoglobin level, % pred.—percentage of predicted value.

**Table 9:** Changes in cardiac 123I-MIBG imaging from baseline to 12-week follow-up.

N = 16	Baseline Median (IQR)	12 Weeks Follow-Up Median (IQR)	*p*-Value *
HMR (early)	1.86 (0.43)	2.03 (0.43)	*p* = 0.252
HMR (late)	1.88 (0.37)	2 (0.41)	*p* = 0.026
WR (%)	31.65 (30.8)	19.5 (21.85)	*p* = 0.464
LV TDS (early)	7 (14)	8 (11.25)	*p* = 0.72
LV TDS (late)	21 (16)	14.5 (15.75)	*p* = 0.502

* *p*-value ≤ 0.05 indicates statistically significant results. Abbreviations: IQR—interquartile range, HMR—heart-to-mediastinum ratio, WR—washout rate, LV—left ventricular, TDS—total defect score.

## Data Availability

The original contributions presented in the study are included in the article, further inquiries can be directed to the corresponding author.
